# The Effect of Reduced Graphene Oxide-Coated Biphasic Calcium Phosphate Bone Graft Material on Osteogenesis

**DOI:** 10.3390/ijms18081725

**Published:** 2017-08-08

**Authors:** Jeong-Woo Kim, Yong Cheol Shin, Jin-Ju Lee, Eun-Bin Bae, Young-Chan Jeon, Chang-Mo Jeong, Mi-Jung Yun, So-Hyoun Lee, Dong-Wook Han, Jung-Bo Huh

**Affiliations:** 1 Department of Prosthodontics, Dental Research Institute, Institute of Translational Dental Sciences, BK21 PLUS Project, School of Dentistry, Pusan National University, Yangsan 50612, Korea; niceguy9790@hanmail.net (J.-W.K.); ljju1112@hanmail.net (J.-J.L.); 0228dmqls@hanmail.net (E.-B.B.); jeonyc@paran.com (Y.-C.J.); cmjeong@pusan.ac.kr (C.-M.J.); p-venus79@hanmail.net (M.-J.Y.); romilove7@hanmail.net (S.-H.L.); 2 Department of CognoMechatronics Engineering, Pusan National University, Busan 46241, Korea; choel15@naver.com (Y.C.S.); nanohan@pusan.ac.kr (D.-W.H.)

**Keywords:** reduced graphene oxide, biphasic calcium phosphate, bone regeneration, concentration, rat

## Abstract

This study was conducted to evaluate the effect of biphasic calcium phosphate (BCP) coated with reduced graphene oxide (rGO) as bone graft materials on bone regeneration. The rGO-coated BCP bone graft material was fabricatied by mixing rGO and BCP at various concentrations. The surface charge of rGO-coated BCP was measured to be −14.43 mV, which formed a static electrostatic interaction. Cell viabilities were significantly diminished at higher concentrations of ≥100 μg/mL. The calvarial defects of 48 rats were implanted rGO-coated BCPs at a weight ratio of 2:1000 (rGO2), 4:1000 (rGO4), and 10:1000 (rGO10), repectively. BCP was used as a control group. The micro-CT and histological analysis were performed to evaluate new bone formation at 2 and 8 weeks after surgery. The results showed that the new bone volume (mm^3^) was significantly higher in the experimental groups than in the control group. Histological analysis showed that new bone areas (%) were significantly higher in the rGO2 and rGO10 than in the control, and significantly higher in rGO4 than in the rGO2 and rGO10. Conclusively, the rGO-coated BCP was found to be effective on osteogenesis and the concentration of the composite was an important factor.

## 1. Introduction

Graft materials for alveolar bone augmentation can be classified based on the source of the bone as: autogenous, allogeneic, xenogeneic, or alloplastic bone grafts [1]. Autogenous bone grafts are the recognized gold standard, as they encourage osteogenesis and minimize cellular and humoral immune reactions [2]. However, the availability of autogenous bone is limited and harvesting requires additional surgery [3]. Allogeneic bone grafts are not limited with respect to the amount of bone harvested and do not require second surgery, but they do present the risk of disease transmission and raise ethical issues [4]. Xenogeneic bone grafts are easily obtained and manufactured, but they pose infection risks and have low osteoinductive capacities [5]. On the other hand, alloplastic bone grafts, like xenogeneic bone grafts, are readily available but raise concerns about inflammatory reactions and have poor osteoinductive potentials [6].

Alloplastic bone grafts are derived from non-living materials and are manufactured industrially. These grafts are composed of hydroxyapatite (HA) or β-tricalcium phosphate (β-TCP) [7]. Biphasic calcium phosphate (BCP, a mixture of hydroxyapatite and β-tricalcium phosphate) is used widely to control resorption rate and provide space maintenance [8]. Alloplastic bone can be manufactured without restriction and poses no risks of infection or immune response, but its osteoinductive potential is poor [6]. As a result, many studies have been conducted to enhance the bone regeneration performance of alloplastic grafts [9,10,11,12,13,14]. Bone morphogenetic protein-2 and -3 (BMP-2 and -3), β-glycerophosphate, and ascorbate are known osteogenic differentiation-inducing agents [10,11].

Nowdays, graphene is being studied as a means of enhancing bone regeneration [15,16]. At the atomic level, graphene is a flat monolayer of carbon atoms tightly packed into a two-dimensional (2D) honeycomb lattice [17]. Graphene has unique mechanical, electrical, thermal, and optical properties, and thus, has applications in many fields, such as, electronics, semiconductors, energy production, polymer science, and biotechnology [18]. The potential of graphene and its derivatives to enhance stem cell differentiation is of particular interest. To enhance stem cell differentiation, graphene and its derivatives function as planar culture platforms to differentiate stem cells towards osteogenic, neurogenic, myogenic, and chondrogenic lineages [19,20,21,22,23,24]. There are three types of graphene: pristine graphene, graphene oxide (GO), and reduced graphene oxide (rGO). Pristine graphene is a thin layer of pure carbon produced mainly by chemical vapor deposition (CVD), by the exfoliation of graphite [25]. GO is a hydrophilic oxidized form of graphene, and as its name implies, contains oxygen-containing groups [26]. rGO is obtained from reduction of GO, which removes oxygen-containing groups [27]. Of the three different forms of graphene, rGO has been focused on more so as a biomaterial that enhances cellular behaviors, because rGO is biocompatible and has some reactive functional groups [27,28,29]. Furthermore, the toxicity of GO can be minimized by controlling GO reduction [28]. In one in vitro study, graphene was found to enhance the adherence and growth of mesenchymal stem cells and osteoblasts [30], and in an animal study, rGO hydroxyapatite nanocomposites enhanced osteogenesis, though the particle size of hydroxyapatite used was considerably smaller than that typical of clinical bone graft materials [31].

Accordingly, using a combination of graphene and alloplastic bone graft in dental clinics was considered to enhance bone regeneration. In the present study, a biphasic calcium phosphate bone graft material was mixed in different ratios with rGO and these materials were evaluated to investigate the effects of rGO on bone regeneration.

## 2. Results

### 2.1. In Vitro Results

#### 2.1.1. Characterizations of Reduced Graphene Oxide (rGO)-Coated Biphasic Calcium Phosphate (BCP) Bone Graft Materials

**Figure 1:**
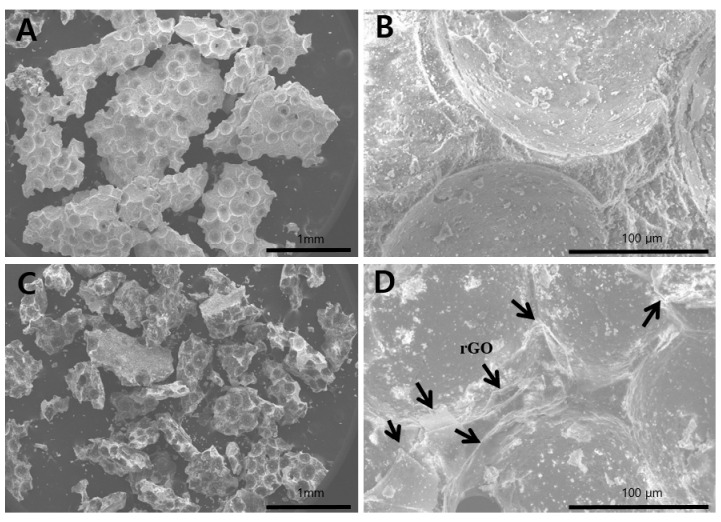
Field emission scanning electron microscope (FE-SEM) images of biphasic calcium phosphate (BCP) microparticles (magnification: 30× (**A**); 500× (**B**)) and reduced graphene oxide (rGO)-coated BCP bone graft materials (magnification: 30× (**C**); 500× (**D**)). Arrows indicate the rGO nanoplatelets.

The surface morphology of a BCP microparticle (Bio-C) and of a rGO-coated BCP bone graft material are shown in Figure 1. A field emission scanning electron microscope (FE-SEM, Hitachi S-4700, Hitachi, Tokyo, Japan) showed rGO-coated BCP microparticles had an irregular granule-like shape with diameters of several microns, and were partly covered by an interconnected network of rGO nanoplatelets (Figure 1D).

**Figure 2:**
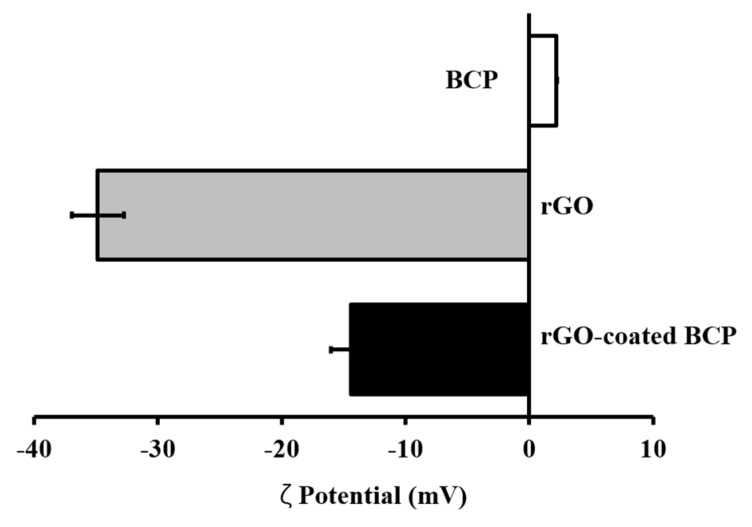
The surface charge of BCP, RGO, and rGO-coated BCP by the ζ potential analysis.

The ζ potential analysis showed that BCP microparticles in deionized water (pH 7.0) were charged at about +2.17 mV, whereas rGO nanoplatelets were charged at about −34.5 mV (Figure 2). The surface charge of rGO-coated BCP was be around −14.43 mV. These results indicate that rGO-coated BCP bone graft material was probably formed by electrostatic interactions between BCP microparticles and rGO nanoplatelets.

**Figure 3:**
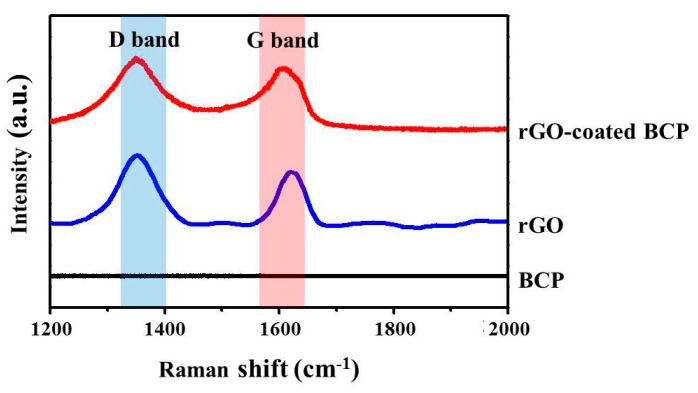
The Raman spectrum of rGO-coated BCP were observed at 1350 and 1600 cm^−1^, corresponding to the D and G bands of rGO nanoplatelets.

The Raman spectra of rGO-coated BCP and BCP are presented in Figure 3. In the Raman spectrum of rGO-coated BCP, characteristic bands of rGO nanoplatelets, corresponding to the D and G bands of rGO nanoplatelets, were observed at ~1350 and ~1600 cm^−1^, respectively. On the other hand, no notable peak was observed in the Raman spectrum of BCP microparticles [32,33].

#### 2.1.2. Cytotoxicity of rGO Nanoplatelets

**Figure 4:**
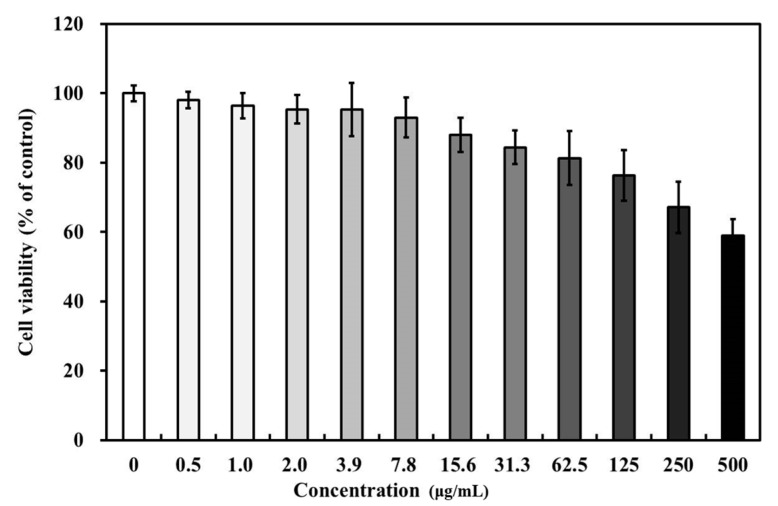
The viability of MC3T3-E1 osteoblasts according to increasing rGO nanoplatelet concentration (μg/mL).

The viabilities of MC3T3-E1 osteoblasts decreased on increasing rGO nanoplatelet concentration (Figure 4). Cell viabilities were slightly affected at concentrations <62.5 μg/mL, but were significantly (*p* < 0.05) diminished at higher concentrations (≥100 μg/mL). At an rGO nanoplatelet concentration of 500 μg/mL, MC3T3-E1 osteoblast viability fell to ~40% of non-treated controls.

### 2.2. In Vivo Results

#### 2.2.1. Clinical Findings

Forty-eight male Sprague–Dawley rats survived the experimental procedure, and all 48 defects were harvested without problem. During the treatment period after surgery, no collagen membrane was exposed or lost, and no other abnormal findings, such as infection or inflammation, were observed.

#### 2.2.2. Micro-Computed Tomography (Micro-CT) Findings

**Figure 5:**
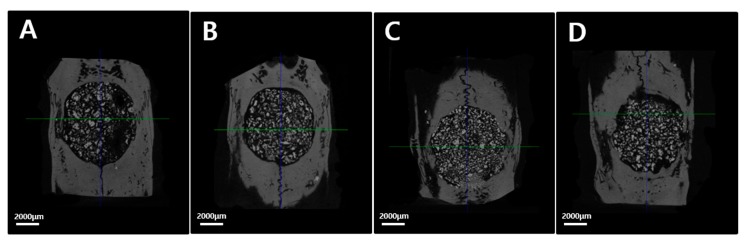
Micro-computed tomographic images. (**A**) Control group; (**B**) rGO2 group; (**C**) rGO4 group; (**D**) rGO10 group.

**Figure 6:**
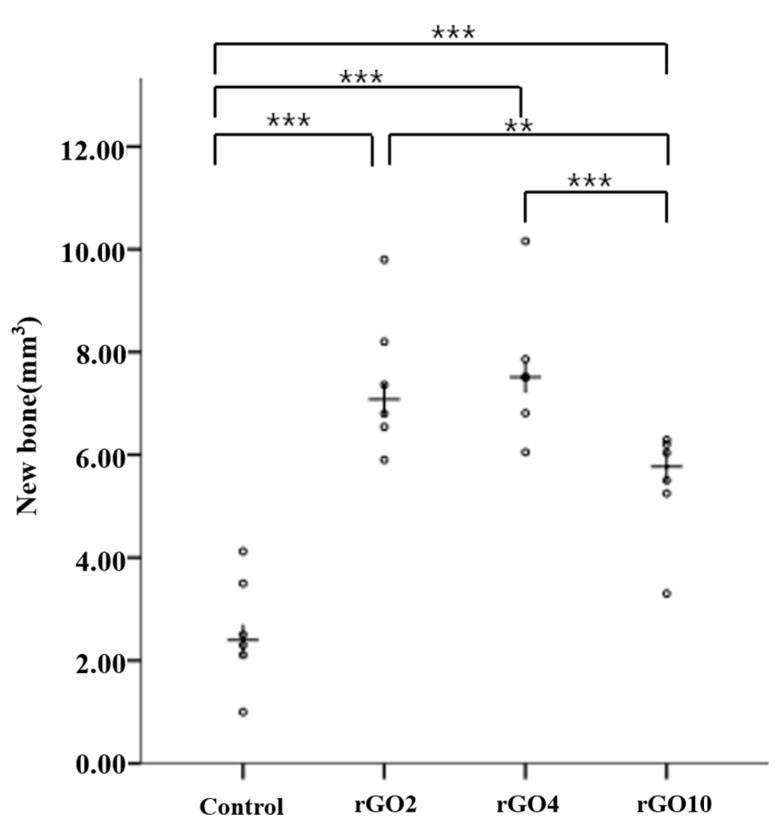
Scatter plot and median values (the crosses) of new bone volumes (mm^3^) (** *p* < 0.01, *** *p* < 0.001).

Total volumes of new bone within regions of interest (ROIs) were measured using the micro-CT scanner at 8 weeks after surgery. Micro-CT images showed the bone graft material was well positioned in defects (Figure 5). No macroscopic difference was observed between the control and experimental groups. New bone volumes, determined by micro-CT, are summarized in Table 1 and Figure 6. At 8 weeks after surgery, mean (±SD) new bone volumes (mm^3^) in the control, rGO2, rGO4, and rGO10 groups was 2.59 ± 1.10, 7.43 ± 1.40, 7.65 ± 1.39, and 5.43 ± 1.12, respectively, and new bone volumes in the experimental groups were significantly different (*p* < 0.001). Furthermore, new bone volume was significantly greater in the three experimental groups than in the control group at week 8 (*p* < 0.05). The rGO4 group exhibited the greatest amount of new bone volume, and the control group the least. The three experimental groups tended to have significantly greater new bone volume values than the control group.

#### 2.2.3. Histologic Findings

No abnormal findings, such as inflammation, were noted in any group. In the three experimental groups, bone graft materials were well positioned in calvarial defect sites, presumably due to the presence of the collagen membrane.

**Figure 7:**
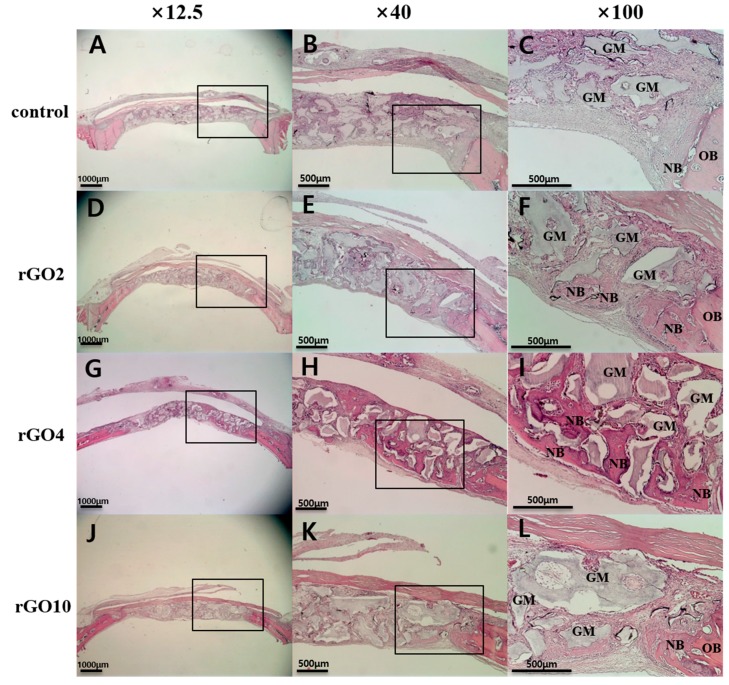
Histological view of hematoxylin and eosin (H&E) stained specimens obtained at 2 weeks after surgery. Black box is the area of interest. (**A**–**C**) Control group; (**D**–**F**) rGO2 group; (**G**–**I**) rGO4 group; (**J**–**L**) rGO10 group; OB: old bone, NB: new bone, GM: bone graft material (Original magnifications: 12.5× (**A**,**D**,**G**,**J**), 40× (**B**,**E**,**H**,**K**), 100× (**C**,**F**,**I**,**L**)).

At 2 weeks after surgery (Figure 7), the early phase of new bone formation was observed around existing bone in calvarial defects in the control and experimental groups, but most of the new bone tissues were immature. In the control group, large quantities of fibrous and connective tissues were observed without inflammation in entire calvarial defect sites. Whereas in the rGO4 group, a large quantity of new bone formation from existing bone around the bone graft material was observed. As compared with the control group, the bone formation in the three rGO groups was enhanced.

**Figure 8:**
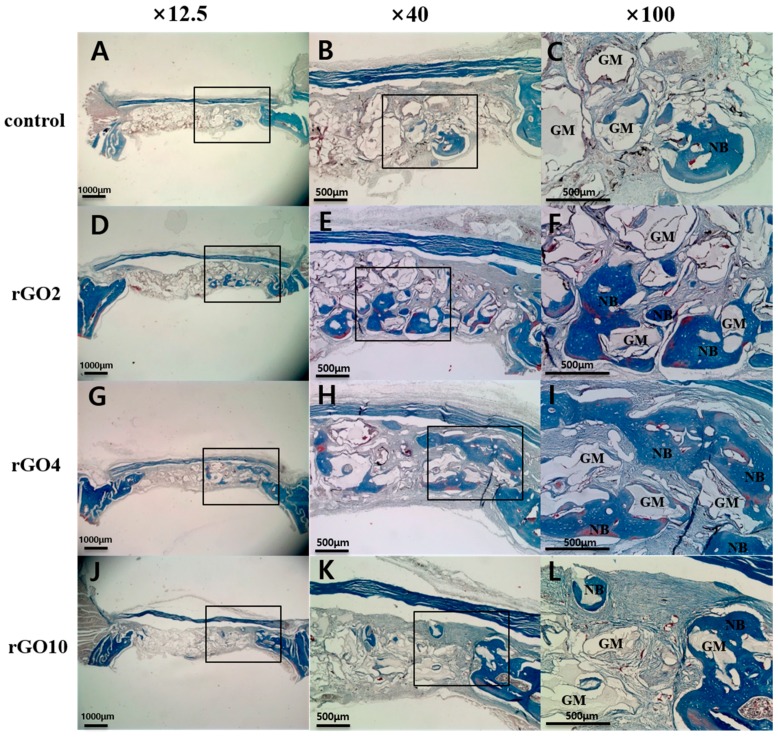
Histological view of Masson’s trichrome stained sections at 8 weeks after surgery. Black box is the area of interest. (**A**–**C**) Control group; (**D**–**F**) rGO2 group; (**G**–**I**) rGO4 group; (**J**–**L**) rGO10 group; OB: old bone, NB: new bone, GM: bone graft material (Original magnifications: 12.5× (**A**,**D**,**G**,**J**), 40× (**B**,**E**,**H**,**K**), 100× (**C**,**F**,**I**,**L**)).

At 8 weeks after surgery (Figure 8), new bone formation and calcification were observed in the control and experimental groups. In most specimens, new bone formation was observed around existing bone and surrounded bone graft materials. At 8 weeks, in the rGO4 group, a large quantity of new bone from existing old bone surrounded bone graft materials. As compared with the control group, all three rGO groups showed obviously more new bone formation at 8 weeks after surgery.

#### 2.2.4. Histometric Findings

**Figure 9:**
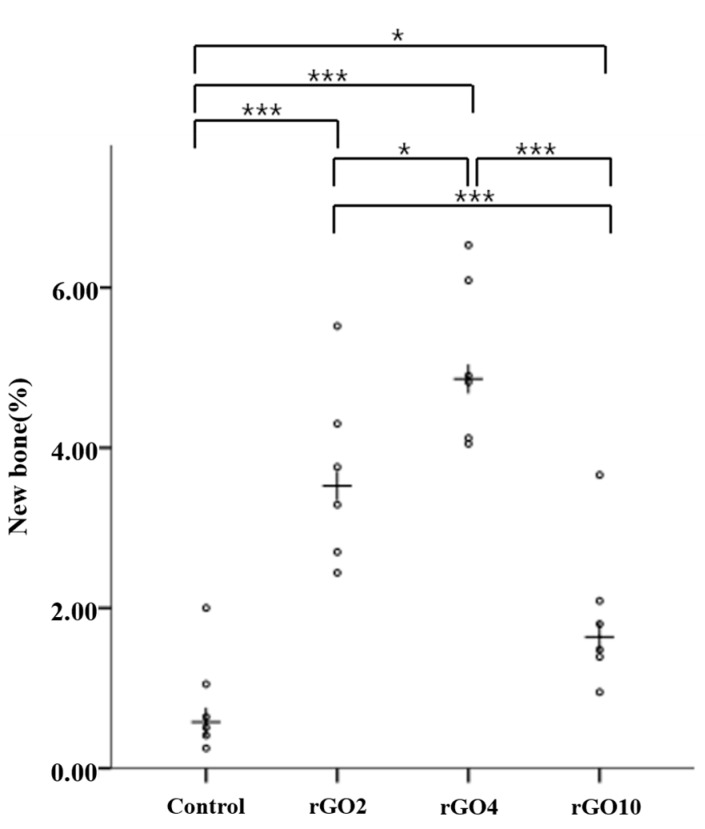
Scatter plot and medians (crosses) of new bone area percentages (%) at 2 weeks after surgery (* *p* < 0.05, *** *p* < 0.001).

**Figure 10:**
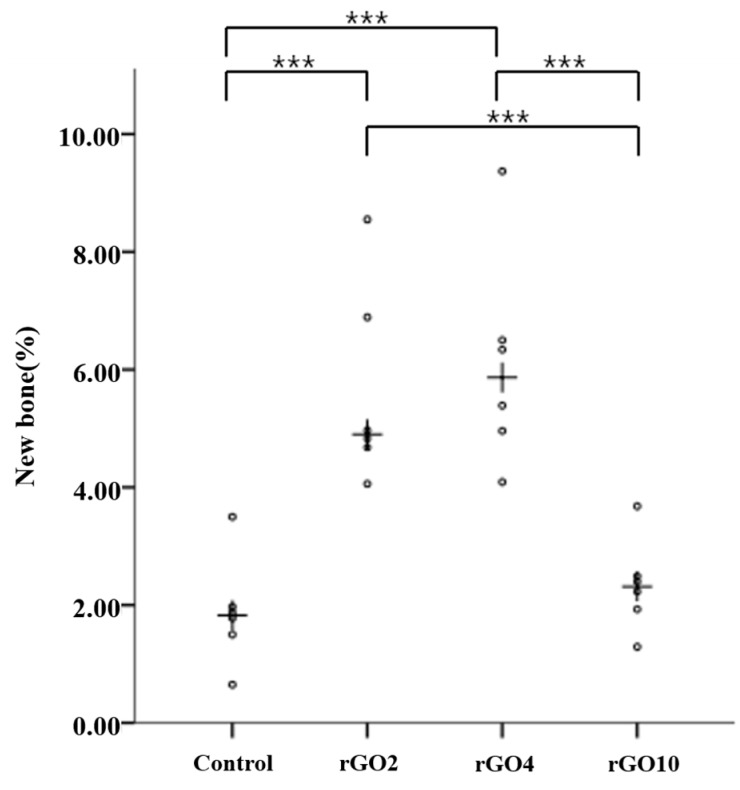
Scatter plot and medians (crosses) of new bone area percentages (%) at 8 weeks after surgery (*** *p* < 0.001).

Histometric measurements are summarized in Table 2 and Figure 9 and Figure 10. Differences in new bone area percentages (%) were significant between groups (*p* < 0.001). 

At 2 weeks, mean (± SD) new bone area percentages (%) in the control, rGO2, rGO4, and rGO10 groups were 0.81 ± 0.64, 3.67 ± 1.13, 5.08 ± 1.02, and 1.90 ± 0.95, respectively. The rGO4 group had the highest new bone area percentage (%), and the control group showed the lowest. New bone area percentages were significantly higher in the experimental groups than in the control group at 2 weeks (*p* < 0.05).

At 8 weeks, mean (±SD) new bone area percentages (%) in the control, rGO2, rGO4, and rGO10 groups were 1.88 ± 0.93, 5.66 ± 1.71, 6.11 ± 1.83, and 2.34 ± 0.79, respectively. New bone area percentages were significantly higher in the rGO2 and rGO10 experimental groups than in the control group, and significantly higher in rGO4 group than in the rGO2 and rGO10 groups (*p* < 0.05).

## 3. Discussion

Graphene is composed of a single layer of carbon atoms that are tightly bonded in a hexagonal honeycomb lattice, which is the basic building block of all graphitic materials [17]. Graphene is used in many different biomedical fields, such as, drug delivery, gene delivery, biosensing, phototherapy for cancer, and bioimaging [34,35,36,37,38,39]. Graphene and its derivates allow the attachment of stem cells and stimulate their growth and their differentiation to the osteogenic lineage [40]. According to Kalbacova, graphene enhances the adherence and growth of mesenchymal stem cells and osteoblasts, but is not cytotoxic to these cells, and also has the potential to differentiate mesenchymal stem cells to the osteoblastic lineage [30]. Shin et al. [41] reported that rGO hydroxyapatite nanocomposites function as effective bone fillers that stimulate the osteogenesis of preosteoblasts.

In this study, BCP was coated at different levels with rGO, and this was confirmed by FE-SEM. In FE-SEM images, BCP was partially covered and interconnected by an rGO network. Previous studies have reported that osteoblasts adhered well to and proliferated on rGO- or graphene-hydroxyapatite hybrid materials, which suggests that these materials induce the three dimensional (3D) adhesion of osteoblast cells and maintain cell viability by providing a microenvironment similar to that found in vivo [42]. ζ potential analysis was used to determine surface potentials, and the results obtained suggested that rGO-coated BCP composite had a stable surface and a surface charge of −14.43 mV, which indicated rGO-coated BCP bone graft material was formed by electrostatic interactions between BCP and rGO. In the Raman spectrum of the rGO-coated materials, the intensity ratio of the D and G bands (ID/IG) is commonly used to characterize graphene and its derivatives [43]. The G band is attributed to the in-plane stretching vibration of graphene, while the D band represents structural defects in sp2-bonded carbon domains [44,45]. Therefore, the ID/IG value of rGO is larger than 1. As shown in Figure 3, the ID/IG value of rGO-coated BCP composite was larger than 1 (~1.05), indicating that the rGO nanoplatelets were successfully prepared, and the structure of rGO nanoplatelets were maintained in the rGO-coated BCP. Therefore, considering the FE-SEM images (Figure 1) together with Raman spectra (Figure 3), it is indicated that the rGO-coated BCP was successfully prepared. The influence of rGO on cell growth is largely dependent on its size, structure, and concentration [46,47,48,49]. In the present study, the cytotoxicity of rGO was evaluated using a cell counting kit-8 (CCK-8) assay, which is based on mitochondrial activity. According to a previous study, the cytotoxicity of rGO involves the generation of an oxidative stress response [46]. rGO has been reported to exhibit dose-dependent cytotoxicity but showed no apparent cytotoxicity at low concentration [47,48]. In the present study, cell viability was significantly decreased at rGO concentrations >100 µg/mL, but was maintained above 80% at concentrations <62.5 µg/mL. These results suggest rGO has no harmful effects and is non-cytotoxic at concentrations <62.5 µg/mL.

At 8 weeks after surgery, total volumes of new bone (mm^3^) were measured by micro-CT, and it showed that the rGO-coated BCP groups exhibited significantly more new bone formation than the control group. The rGO4 group involved the use of a rGO and BCP 4:1000 mixture and it showed the greatest total volume of new bone. Micro-CT and histometric analysis showed that the rGO4 group showed the highest new bone area percentage, and somewhat surprisingly the rGO10 group had a lower new bone area percentage than the rGO2 group. These results suggest as the percentage of rGO is increased, bone regeneration ability is enhanced, but when the rGO percentage exceeds a certain threshold level, rGO cytotoxicity decreases osteoblast viability.

The physical and chemical characteristics of graphene are presumably responsible for its enhancement of osteogenic differentiation. In particular, the wrinkles and ripples present on graphene surfaces may affect osteogenic differentiation [40,50], as these features assist cell anchorage and increase cytoskeletal tension [50,51]. In addition, graphene can adsorb proteins (β-glycerophosphate) and biomolecules (dexamethasone) and these could promote cell differentiation [38].

In this study, we found that rGO-coated BCP bone graft material accelerated new bone formation. We suggest further studies be undertaken to determine the optimal concentration of rGO.

## 4. Materials and Methods

### 4.1. In Vitro Study

#### 4.1.1. Preparation of rGO Nanoplatelets

**Figure 11:**
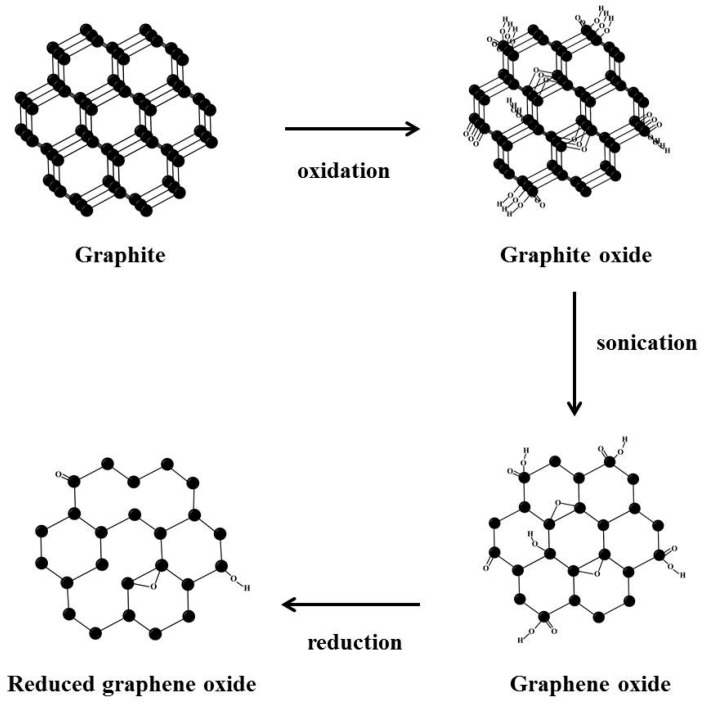
Schematic images of the process used to produce reduced graphene oxide (rGO).

GO was prepared from flake graphite using the modified Hummers and Offeman method (Figure 11) [41,52]. A small amount of graphite flakes (Grade 1721, Asbury Carbon, Asbury, NJ, USA) was placed into a glass beaker and heated for 10 s in a microwave oven. Separately, H_2_SO_4_ (50 mM) was added to a flask, which was then placed in a cold water bath (0 °C) with stirring. Microwave treated graphite flakes (2 g) were then slowly added to this flask, and this was followed by slowly adding 6 g KMnO_4_ powder. This mixture was then heated for 2 h at 35 °C with stirring. Deionized water was then slowly added to the mixture, which was maintained at a temperature of <70 °C. To eliminate the KMnO_4_, H_2_O_2_ (30 wt %) was then added gradually. At this stage the color of the mixture changed from a dark brown to a brilliant yellow. The mixture was filtered several times and diluted with deionized water to completely remove acid until its pH reached 6. GO was obtained by drying the resulting suspension for 12 h. To prepare rGO nanoplatelets, the prepared GO suspension (1 g in 1 L deionized water) was sonicated for 2 h, and then 10 mL hydrazine hydrate (N_2_H_4_·H_2_O) was added to the GO suspension. The reaction was conducted at 100 °C for 24 h, and the suspension obtained was filtered several times and washed with ethanol–water solutions. rGO nanoplatelets were prepared by drying under vacuum conditions at 80 °C for 12 h [52].

#### 4.1.2. Preparation of rGO-coated BCP Bone Graft Material

The BCP microparticles (MP; Bio-C, Cowellmedi, Seoul) used in this study were a mixture of HA and β-TCP (3:7 by weight). To prepare rGO-coated BCP graft material, as-prepared rGO in deionized water was sonicated for 2 h, and then mixed with BCP suspended in deionized water at rGO to BCP weight ratios of 2:1000, 4:1000, or 10:1000. The rGO-coated BCP graft material was obtained by vigorously mixing colloidal dispersions of rGO nanoplatelets and BCP microparticles for 10 min and slow air-drying at room temperature overnight.

#### 4.1.3. Characterizations of the rGO-Coated BCP Bone Graft Materials

The morphologies of rGO-coated BCPs were studied using a FE-SEM (Hitachi S-4700, Hitachi, Tokyo, Japan) at an accelerating voltage of 5 kV. The surface potentials of the rGO-coated BCPs were obtained using a Zetasizer (Malvern Instruments, Nano ZS, Worcestershire, UK). The Raman spectra of rGO-coated BCP, rGO nanoplatelets, and BCP were obtained by a Raman spectrometer (Micro Raman PL Mapping System, Dongwoo Optron Co., Kwangju, Korea) with excitation a 514.5 nm using an Ar-ion laser at a radiant power of 5 mW.

#### 4.1.4. Cytotoxicity of rGO Nanoplatelets

A murine preosteoblastic cell line (MC3T3-E1 cells from C57BL/6 mouse calvaria) was purchased from the American Type Culture Collection (CRL-2593™; ATCC, Rockville, MD, USA). This cell-line is an established osteoblast model and has been widely used to investigate osteogenesis and bone formation [53,54]. Cells were maintained in complete α-minimum essential medium supplemented with 10% fetal bovine serum and 1% antibiotic antimycotic solution (all from Sigma-Aldrich, St Louis, MO, USA) in a humid 5% CO_2_ atmosphere at 37 °C. The cytotoxic effects of rGO nanoplatelets on MC3T3-E1 cells were assessed using a cell counting kit-8 (CCK-8) (Dojindo, Kumamoto, Japan), according to the manufacturer’s instructions. Numbers of viable cells were found to be directly proportional to the metabolic reaction products obtained in the CCK-8 assay. Briefly, the MC3T3-E1 cells were seeded at a density of 5 × 10^4^ cells/mL on 24-well plates and incubated for 24 h. The rGO nanoplatelets were then added at concentrations from 0 to 500 μg/mL, and the cells were further cultured for 24 h. Subsequently, the cells were incubated with a CCK-8 solution for the last 2 h of the culture period (24 h) at 37 °C in the dark. Absorbances were measured at 450 nm using an ELISA reader (SpectraMax 340, Molecular Device Co., Sunnyvale, CA, USA).

### 4.2. In Vivo Study

#### 4.2.1. Experimental Animals and Design

Forty-eight 12- to 13-week-old male Sprague–Dawley rats (250–300 g) were used in this study. Before starting, rats were allowed and a minimum adaption period of 7 days. During all procedures, rats were housed in plastic cages (maximum of 3 rats per cage) under controlled conditions (25 ± 1 °C and RH 55 ± 7%). All animals had access to a rodent diet and water ad libitum. The experimental protocol was approved beforehand by the Pusan National University Institutional Animal Care and Use Committee (PNU-2016-1407, 7 January 2016) and performed at the Laboratory Animal Resource Center of Pusan National University.

The animals were divided into four groups depending on bone graft material used.

1. Control group (*n* = 12)
  : only BCP;
2. rGO2 group (*n* = 12)
  : a mixture of rGO and BCP at a ratio of 2:1000 (Concentration of rGO: 28 μg/mL);
3. rGO4 group (*n* = 12)
  : a mixture of rGO and BCP at a ratio of 4:1000 (Concentration of rGO: 56 μg/mL);
4. rGO10 group (*n* = 12)
  : a mixture of rGO and BCP at a ratio of 10:1000 (Concentration of rGO: 140 μg/mL).

#### 4.2.2. Surgical Procedures

All surgical procedures were performed under general anesthesia. A mixture of tiletamine-zolazepam (Zoletil 50, Virbac, Carros, France) and xylazine (Rompun, Bayer Korea, Seoul) at 6:4 was anesthetized by intramuscular injection into animals. 0.2 mL of anesthetic per rat was used [55]. Surgical sites on rat crania were shaved and disinfected with alcohol and povidone-iodine (Betadine, Korea Pharma Co., Seoul, Korea). Local anesthesia was performed using 2% lidocaine containing 1:100,000 epinephrine (Yu-Han Co., Gunpo, Korea) at surgical sites.

**Figure 12:**
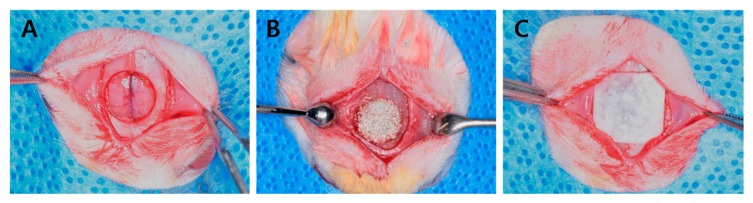
Surgical procedures. (**A**) A defect was formed in the middle of calvaria using a trephine bur; (**B**) Bone graft material was placed; (**C**) A collagen membrane was placed on the defect.

A 15-mm incision was made at midline and the skin and periosteum were dissected to expose parietal bones. An 8-mm diameter bone defect was then formed in the middle of calvaria using a trephine bur (external diameter 8 mm, 3i Implant Innovation, Palm Beach Garden, FL, USA) under saline irrigation. In the control group, 0.03 ± 0.002 g of Bio-C was placed on the calvarial defect using a micro spoon (Karl Hammacher GmbH Co., Cologne, Germany), and in the three experimental groups, the same amount of rGO-coated BCP was placed in the same manner. After completing the bone grafting, a collagen membrane (GCM2030, GENOSS, Suwon, Korea) was cut into square shape (10 mm × 10 mm) and used to cover the defect (Figure 12). Periosteum at the incision site was sutured using a 4-0 absorbable suture (Vicryl, Ethicon, Somerville, NJ, USA) and skin was sutured with 4-0 black silk.

Animals were allowed to recover for 2 or 8 weeks after surgery. In the three experimental groups, animals were sacrificed by CO_2_ inhalation at 2 or 8 weeks after surgery. To collect specimens, skin was removed, and defect sites were harvested with surrounding bone. Collected specimens were immediately immersed in neutral buffered formalin (Sigma-Aldrich) for 2 weeks and prepared for micro-computed tomographic (micro-CT) and histomorphometric analyses.

#### 4.2.3. Micro-Computed Tomography Analysis

**Figure 13:**
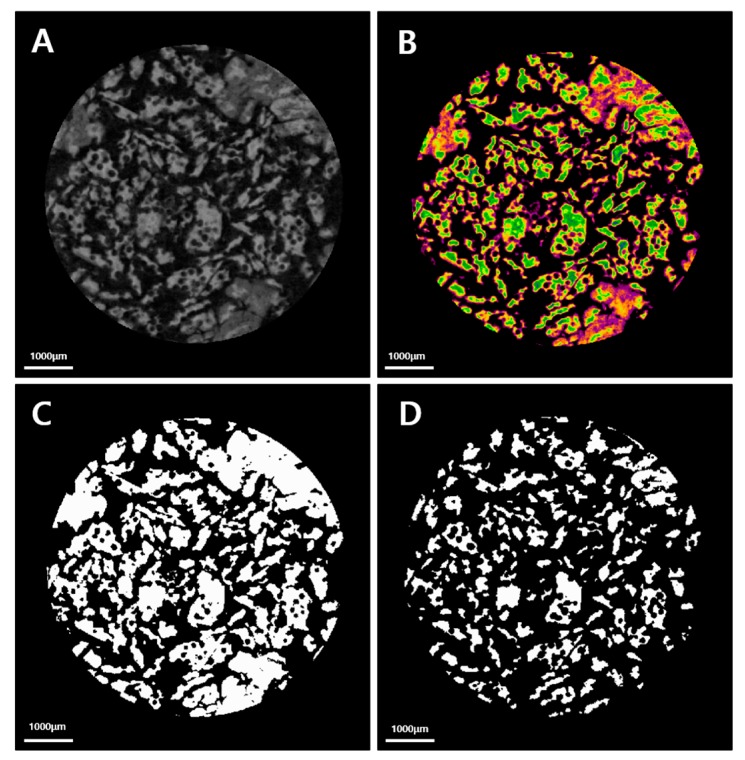
Micro-computed tomographic (CT) images of regions of interest. (**A**) Reconstructed image; (**B**) Color image (yellow and green-bone graft material and orange and purple-new bone); (**C**) Total bone (bone graft material and new bone) image; (**D**) Bone graft material image.

Harvested calvaria were scanned using a micro-CT scanner (Skyscan-1173, version 1.6, Bruker-CT Co., Kontich, Belgium) at 8 weeks after surgery (Figure 13). Harvested calvaria were wrapped in film (Parafilm M, Bemis Co., Neenah, WI, USA) to prevent evaporation of fixative solution during scans. Scanning was performed at 130 kV, 60 μA, at a pixel resolution of 7.10 μm using a bromine filter (0.25 mm). The Nrecon reconstruction program (version 1.6.10.1, Bruker-CT Co., Kontich, Belgium) was used to reconstruct images. Identical parameters were used for all specimens. A cylindrical region of interest (ROI; 8 mm diameter; the same size as the original defect) was established, and total volume of new bone volume (NBV; mm^3^), that is, the volume occupied by new bone within the region of interest, was measured.

#### 4.2.4. Histologic and Histometric Analysis

**Figure 14:**
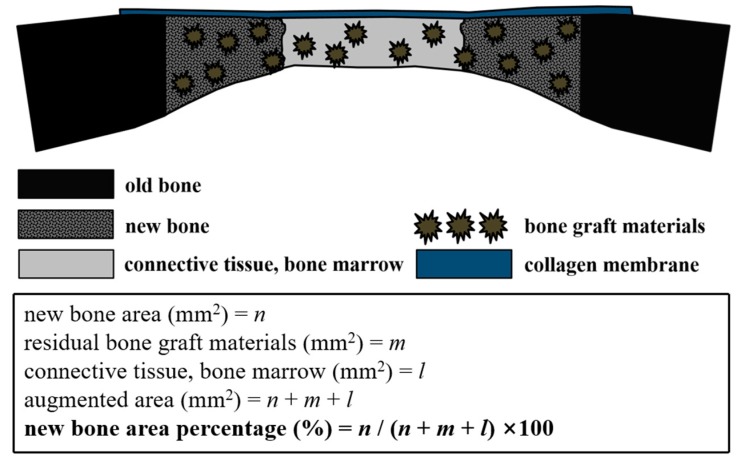
Schematic illustration of the histometric analysis.

Specimens were decalcified in 10% ethylenediaminetetraacetic acid (EDTA) solution (pH 8.0) for 2 weeks at 4 °C. After dehydration, specimens were embedded in paraffin, and sectioned longitudinally at 4 µm through the largest defect diameter using a microtome. Slides were stained with hematoxylin-eosin and Masson’s trichrome stain for histologic and histometric analyses to observe newly regenerated bone tissues, and photographed under an optical microscope (BX51, OLYMPUS, Tokyo, Japan) connected to a computer. Captured images were analyzed using an image analysis program (i-solution ver. 8.1, IMT, Vancouver, BC, Canada). For histologic and histometric analyses, specimen images were observed at magnifications of 12.5×, 40×, and 100×. Histologic and histometric analyses were performed by the same blinded investigator. Total area of new bone was defined as the area occupied by new bone within a defect (Figure 14), and new bone area (%) (NBA %) was defined as the area occupied by new bone expressed as a percentage of total defect area.

#### 4.2.5. Statistical Analysis

Results are expressed as means, standard deviations, and medians. The analysis was conducted using the statistical software package R ver. 3.1.3 (The R Foundation, Vienna, Austria). To compare group micro-CT and histomorphometric results, we used the non-parametric analysis devised by Brunner & Langer. The statistical significance was accepted for *p* values of <0.05.

## 5. Conclusions

The in vitro results obtained in the present study indicate that reduced graphene oxide-coated biphasic calcium phosphate is a safe and stable bone graft material. The reduced graphene oxide-coated biphasic calcium phosphate enhanced bone regeneration better than biphasic calcium phosphate alone. In addition, new bone formation appeared to be influenced by the concentration of reduced graphene oxide in the composite. We suggest further studies be undertaken to determine the optimum concentration of reduced graphene oxide in the composite.

## Figures and Tables

**Table 1:** New bone volumes within regions of interest (*n* = 6; mm^3^).

Group	Mean ± SD	Median
Control	2.59 ± 1.10	2.40
rGO2	7.43 ± 1.40	7.08
rGO4	7.65 ± 1.39	7.51
rGO10	5.43 ± 1.12	5.77

**Table 2:** New bone area percentages within the areas of interest (*n* = 6; %).

Week	Group	Mean ± SD	Median
2	Control	0.81 ± 0.64	0.57
	rGO2	3.67 ± 1.13	3.52
	rGO4	5.08 ± 1.02	4.86
	rGO10	1.90 ± 0.95	1.64
8	Control	1.88 ± 0.93	1.83
	rGO2	5.66 ± 1.71	4.90
	rGO4	6.11 ± 1.83	5.87
	rGO10	2.34 ± 0.79	2.31

## Abbreviations

BCP	Biphasic calcium phosphate
rGO	Reduced graphene oxide
HA	Hydroxyapatite
β-TCP	β-Tricalcium phosphate
BMP	Bone morphogenetic protein
2D	Two-dimensional
GO	Graphene oxide
Bio-C	BCP microparticle
FE-SEM	Field emission-scanning electron microscopy
H&E	Hematoxylin and eosin
3D	Three dimensional
ID/IG	Intensity ratio of the D and G bands
micro-CT	Micro-computed tomographic
ROI	Region of interest
NBV	New bone volume
EDTA	Ethylenediaminetetraacetic acid
NBA	New bone area
